# Genomic selection for heterobothriosis resistance concurrent with body size in the tiger pufferfish, *Takifugu rubripes*

**DOI:** 10.1038/s41598-020-77069-z

**Published:** 2020-11-17

**Authors:** Zijie Lin, Sho Hosoya, Mana Sato, Naoki Mizuno, Yuki Kobayashi, Takuya Itou, Kiyoshi Kikuchi

**Affiliations:** 1 Fisheries Laboratory, University of Tokyo, Hamamatsu, Shizuoka 431-0214 Japan; 2 Veterinary Research Center, Nihon University, Fujisawa, Kanagawa 252-0880 Japan

**Keywords:** Agricultural genetics, Animal breeding, Genetic association study, Heritable quantitative trait

## Abstract

Parasite resistance traits in aquaculture species often have moderate heritability, indicating the potential for genetic improvements by selective breeding. However, parasite resistance is often synonymous with an undesirable negative correlation with body size. In this study, we first tested the feasibility of genomic selection (GS) on resistance to heterobothriosis, caused by the monogenean parasite *Heterobothrium okamotoi*, which leads to huge economic losses in aquaculture of the tiger pufferfish *Takifugu rubripes.* Then, using a simulation study, we tested the possibility of simultaneous improvement of parasite resistance, assessed by parasite counts on host fish (HC), and standard length (SL). Each trait showed moderate heritability (square-root transformed HC: *h*^2^ = 0.308 ± 0.123, S.E.; SL: *h*^2^ = 0.405 ± 0.131). The predictive abilities of genomic prediction among 12 models, including genomic Best Linear Unbiased Predictor (GBLUP), Bayesian regressions, and machine learning procedures, were also moderate for both transformed HC (0.248‒0.344) and SL (0.340‒0.481). These results confirmed the feasibility of GS for this trait. Although an undesirable genetic correlation was suggested between transformed HC and SL (*r*_*g*_ = 0.228), the simulation study suggested the desired gains index can help achieve simultaneous genetic improvements in both traits.

## Introduction

Selective breeding is potentially able to boost aquaculture efficiency, now the fastest-growing food production industry^1^. In particular, pedigree-based breeding methods have contributed to aquaculture development by improving economically important traits, as seen in the salmonids and tilapias^2–4^. However, pedigree-based methods have innate drawbacks where it is assumed estimated breeding values (EBVs) of target traits for candidate individuals are the average breeding values of parents, ignoring Mendelian segregation within families^5^. Thus, pedigree-based methods can not differentiate EBVs among full sibs and large-scale pedigrees were needed to evaluate breeding values. On the other hand, molecular markers can be effective in handling Mendelian sampling by capturing genetic variance at DNA levels, i.e., full sibs could have different EBVs. By harnessing whole-genome high-density markers and advanced regression methods, Meuwissen et al.^6^ proposed genomic selection (GS) to estimate the genomic estimated breeding values (GEBVs) of selection candidates. Thanks to the recent advances in genotyping by sequencing technologies, it is now affordable to genotype genome-wide single nucleotide polymorphisms (SNPs) for GS aquaculture breeding programs^7^. As expected, the greater performance of GS over the pedigree-based method in prediction and inbreeding control has been demonstrated by empirical studies using cultured fish populations^8,9^.

The tiger pufferfish *Takifugu rubripes* is a delicacy in Japan and is one of the most valuable marine fish species in Japanese aquaculture, ranking fourth in production value among cultured finfish^10^. To fulfill the growing demand for this species, selective breeding will be a practical approach to boost farming efficiency; however, tiger pufferfish aquaculture has not yet fully applied this technology^11,12^. Apart from growth-related traits, disease resistance should be highly valued in the breeding program, as disease outbreaks easily hamper the aquaculture industry. For instance, heterobothriosis, the gill disease caused by a monogenean parasite *Heterobothrium okamotoi*, severely threatens tiger pufferfish productivity and welfare^13^. The most severe infectious occurs at early phases of production, just after transfer from land-based hatcheries to sea cages^14,15^. These naïve juveniles are afflicted by the parasite, persistently present at oceanic aquaculture sites, resulting in retarded growth and high mortality rate^16^. While the mechanisms of host immune system response to *H. okamotoi* are still unclear^17,18^, host resistance to heterobothriosis is considered polygenic^19^ and it is difficult to apply marker-assisted selection, which has worked well in infectious pancreatic necrosis resistant Atlantic salmon^20^. Recently, the potential of GS for disease resistance has been demonstrated in farmed populations of Atlantic salmon (*Salmo salar*)^21,22^, rainbow trout (*Oncorhynchus mykiss*)^9^, European sea bass (*Dicentrarchus labrax*)^23^, and gilthead seabream (*Sparus aurata*)^24^. As most of the disease resistance traits have moderate or high heritability in fish species^21–25^, GS can also be applied to facilitate heterobothriosis resistance in the tiger pufferfish.

Selecting one quantitative trait may improve or diminish others due to the genetic pleiotropy and/or linkage disequilibrium^26^. For example, the breeding program which improves the resistance to sea lice possibly diminishes growth-related traits in farmed Atlantic salmon^27^. Likewise, improving resistance to *H. okamotoi* may negatively affect growth-related traits in the tiger pufferfish^11^. Thus, simultaneous genetic improvement of resistance to heterobothriosis and body size is most desirable for aquaculture of the tiger pufferfish, although complicated by traits with antagonistic genetic correlations. One of the conventional methods for multiple-trait improvement is the linear selection index (LSI) method developed by Smith and Hazel^28,29^. Net genetic merit (i.e., LSI) of each animal is calculated from each target trait and used for ranking breeding candidates. To maximize the selection response, a general LSI is computed by a linear combination of phenotypes or EBVs and the corresponding coefficients. Extensive LSI methods have been proposed^30^, as determined by the method of coefficient calculation. For instance, the desired gain selection index allows breeders to restrict traits according to the expected change of genetic gain values of traits^31^. In the era of GS, those LSI methods can be directly applied to compute the linear genomic selection index (LGSI), which showed higher efficiency in both simulation and real data, compared to pedigree-based LSI^32^. Although LGSI showed great advantages, successful applications of LGSI still largely depend on the accurate estimation of GEBVs and genetic parameters^33^, which are sensitive to many factors, including the genetic architecture of target traits, population structure, genotyping technologies, etc.^34–36^. Consequently, an LGSI method might have different performances in different cases. Therefore, it is essential to find the optimal strategy incorporating LGSI and examine its performance in each breeding program. The GS breeding simulator will be a practical tool that approximates the real genetic progress by sophisticated modeling of the meiosis and GS procedure at the DNA level^37^. Further, as regards selection targeting disease resistance traits in aquaculture, a recent simulation study of acute hepatopancreatic necrosis disease (AHPND) in shrimp (*Litopenaeus vannamei*) showed that GS was superior to pedigree-based methods^38^. Therefore, with the assistance of simulation, the breeding strategies incorporating LGSI are expected to greatly accelerate the simultaneous genetic improvement of disease resistance and growth-related traits.

In this study, we tested the possibility of GS to improve heterobothriosis resistance of the tiger pufferfish and designed a GS breeding strategy that could improve the resistance trait concurrent with growth-related traits. We initiated artificial infection on cultured tiger pufferfish obtained from wild parents, applied genome-wide association studies (GWAS), and genetic parameter estimation to survey the genetic architecture of target traits. We then examined the possibility of genomic prediction (GP) for both traits by applying 12 different prediction models. Finally, we investigated the optimal breeding strategy incorporating LGSI using a simulation study by comparing six breeding scenarios.

## Results

### Phenotypes

**Figure 1:**
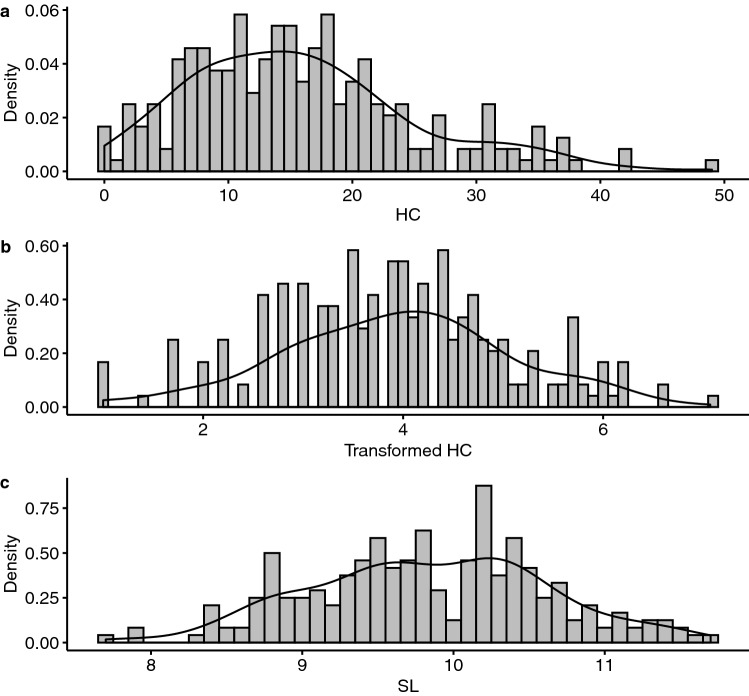
Histograms with the estimated density of phenotypes: (**a**) *Heterobothrium okamotoi* count (HC), (**b**) transformed HC, and (**c**) standard length (SL).

We produced test fishes by artificially crossing 11 wild males and 10 wild females, and subjected 240 4-month-old individuals to an artificial infection for 37 days. Heterobothriosis resistance was evaluated by counting the number of parasites attached to the branchial cavity walls (HC), and the standard length (cm) was measured on each fish (SL). The phenotypic mean was 15.85 (± 9.15 S.D.) for HC and 9.83 (± 0.78 S.D.) for SL (Fig. 1 and Supplementary Table S1). As the plot shows, the distribution of HC was non-normal (Shapiro–Wilk test: *p* = 3.79 × 10^–6^, alpha level = 0.05) while SL approximated a normal distribution (Shapiro–Wilk test: *p* = 0.406, alpha level = 0.05). Therefore, we applied a square-root transformation on (HC + 1), approximating a normal distribution (Shapiro–Wilk test: *p* = 0.235, alpha level = 0.05). Transformed HC was used in the following genetic analysis. Weak but significant phenotypic correlation was observed between HC and SL (Pearson’s *r* analysis: *r* = 0.157, *p* = 0.015; 95% confidence interval: 0.031 ≤ *r* ≤ 0.278).

### Genotyping

We genotyped genome-wide SNPs of each individual using AmpliSeq^39^. The MiSeq sequencing generated an average of 174,870 (± 83,576 S.D.) raw reads per fish. After the quality-trimming step, the mean number of reads for each fish was 161,426 (± 83,576 S.D.) with the mean read length of 124 bp. The survived reads were mapped onto a reference fugu genome (FUGU5/fr3) for SNP calling. Following the quality filtration of SNPs, 6718 putative SNPs were yielded. Missing SNPs were imputed using LinkImputeR^40^. At this imputation step, 11 SNPs were discarded and 6707 imputed SNPs were called for each individual with the imputation accuracy of 0.888.

### Population structure

**Figure 2:**
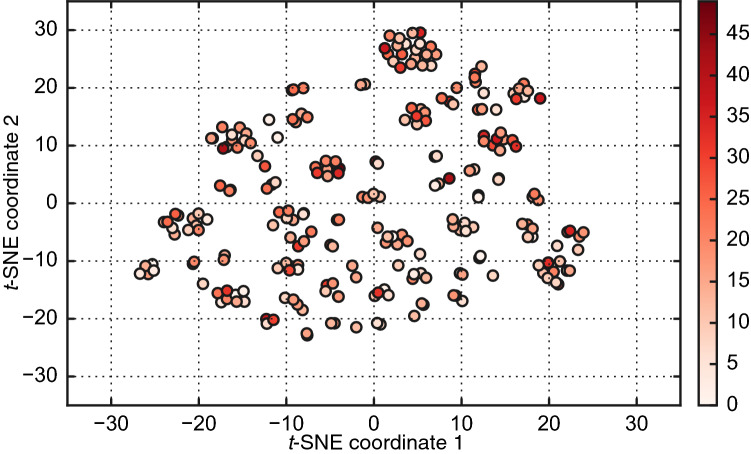
Population structure detected by *t*-SNE analysis based on the genomic SNP data of each individual (filled circle). Filled colors represent *Heterobothrium okamotoi* count of each individual based on the color bar (right panel).

Population structure, which can bias the genetic parameter estimation, was examined by *t*-SNE analysis^41^ based on SNP data (Fig. 2). As seen in the plot, we did not observe clear clusters or strong stratification within the tested samples.

### Heritability and genetic correlation

To investigate the extent of genetic effects on the phenotypic variation, heritability was estimated by a multivariate linear mixed model. Moderate heritability was observed for each trait (transformed HC: *h*^2^ = 0.308 ± 0.123 S.E.; SL: *h*^2^ = 0.405 ± 0.131). With the same model, the strength of the genetic correlation between the transformed HC and SL was also estimated. We detected a moderate antagonistic genetic correlation (*r*_*g*_ = 0.228), where large individuals were suffering from higher parasitic loads. This genetic correlation could be due to the phenotypic correlation, although phenotypic correlation between HC and SL was weak as described above. Therefore, we tested correlation between GEBV for each trait using a univariate linear model (i.e. GBLUP); to predict GEBV for HC, SL was included as the covariate to minimize non-genetic effects from SL. If genetic correlation exists between the two phenotypes, the GEBVs would also show a correlation. We found positive correlation (Pearson’s *r* = 0.252, *p* = 7.67 × 10^–5^). This supports genetic correlation between the two traits.

### Genome-wide association study (GWAS)

**Figure 3:**
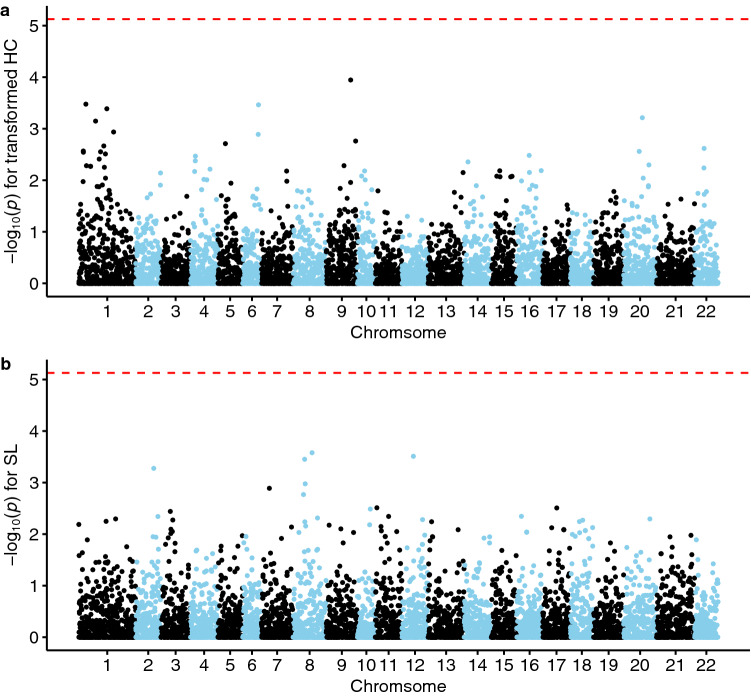
Manhattan plots from genome-wide association study: (**a**) transformed *Heterobothrium okamotoi* count (HC), and (**b**) standard length (SL). Adjacent chromosomes are distinguished by different colors. The X-axis is the physical order of the SNP markers across the 22 chromosomes of *Takifugu rubripes*. The Y-axis represents the negative logarithm of *p*-values (base: 10) for the target trait. Red dashed lines are Bonferroni-corrected significance thresholds of 5.128.

GWAS was applied to detect loci highly associated with transformed HC and SL (Fig. 3). Although none of these loci exceeded the significance threshold of 5.13 (= – log_10_ (0.05/6707)), SNPs with relatively high association were found in the chromosome 1, 6 and 9 for HC (– log_10_(*p*) = 3.48, 3.46 and 3.95, respectively) and in the chromosome 8 and 12 for SL (– log_10_(*p*) = 3.58 and 3.51, respectively).

### Model comparison of genomic prediction

To examine the availability of GS for HC and SL, we applied 12 regression models: i.e., GBLUP, Bayes A, Bayes B, Bayes C, Bayes Ridge, Bayes LASSO, Bayesian reproducing kernel Hilbert space (Bayesian RKHS), support vector machine regression with a linear kernel (SVR-linear), SVR with a poly kernel (SVR-poly), SVR with a radial basis function kernel (SVR-rbf), feedforward neural networks (FNN), and multi-task feedforward neural networks (multi-task FNN). We compared predictive ability defined as Pearson’s *r* between the GEBVs and observed phenotypes by means of a tenfold cross-validation scheme. Predictive abilities for transformed HC ranged from 0.248 to 0.344 under 12 models (Table 1). Among these models, SVR-poly and SVR-rbf models were inferior, while two deep learning models were slightly better. On the contrary, the two SVR based models ranked at the top for prediction of SL, and deep learning models were inferior. Bayes RKHS and GBLUP models showed good performance in both traits.

**Table 1:** Predictive ability (mean ± standard error) on *Heterobothrium okamotoi* count (HC) and standard length (SL) under 12 models: GBLUP, Bayes A, Bayes B, Bayes C, Bayes LASSO, Bayes reproducing kernel Hilbert space (Bayes RKHS), support vector machine with a linear kernel (SVR-linear), SVR with a poly kernel (SVR-poly), SVR with a radial basis function kernel (SVR-rbf), feedforward neural networks (FNN), and multi-task feedforward neural networks (multi-task FNN).

Model	HC	SL
GBLUP	0.307 ± 0.018	0.463 ± 0.018
Bayes A	0.312 ± 0.018	0.461 ± 0.018
Bayes B	0.306 ± 0.018	0.460 ± 0.018
Bayes C	0.307 ± 0.018	0.460 ± 0.018
Bayes LASSO	0.303 ± 0.018	**0.464 ± 0.018**
Bayes ridge	0.304 ± 0.018	0.460 ± 0.018
Bayes RKHS	**0.325 ± 0.019**	0.463 ± 0.018
SVR-linear	0.322 ± 0.017	0.410 ± 0.019
SVR-poly	0.248 ± 0.019	**0.481 ± 0.018**
SVR-rbf	0.249 ± 0.019	**0.475 ± 0.018**
FNN	**0.330 ± 0.018**	0.405 ± 0.017
Multi-task FNN	**0.344 ± 0.019**	0.340 ± 0.022

The top three models for HC and SL are highlighted with bold font.

### Simulation

**Figure 4:**
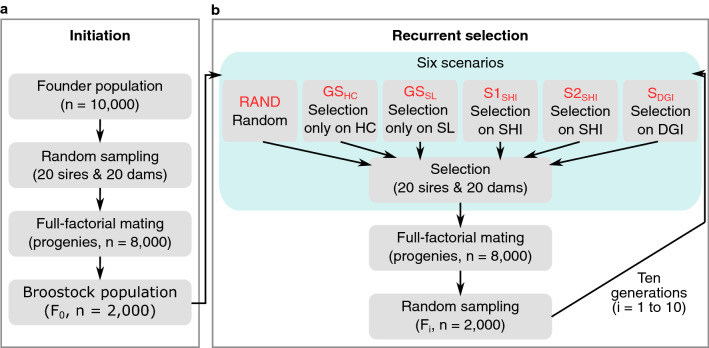
The diagrams of the simulation study. (**a**) The initiation of the breeding program shared among all scenarios. The founder population (n = 10,000) was constructed, and 20 sires and 20 dams were randomly sampled to produce 8000 progenies. Then, 2000 fish were randomly sampled from the progeny pool as the broodstock population (F_0_). (**b**) The workflow of recurrent selection schemes. Parents (20 sires and 20 dams) were selected from F_0_ according to the scenario-specific selection criteria and 8000 progenies were generated. The selection scenarios were: RAND, random selection; GS_HC_, selection on *Heterobothrium okamotoi* counts (HC); GS_SL_, selection on standard length (SL); S1_SHI_ and S2_SHI_, selection based on genomic Smith-Hazel index (SHI); S_DGI_, selection based on the desired gains index (DGI). S1_SHI_ has the same economic weights for both traits, and S2_SHI_ uses the similar vector of economic weights as the vector of desired gains in S_DGI_. Then, random sampling was applied to select 2000 progenies as the broodstock population for the next generation. A total of 10 generations (F_1_ to F_10_) of this process were replicated 50 times.

**Figure 5:**
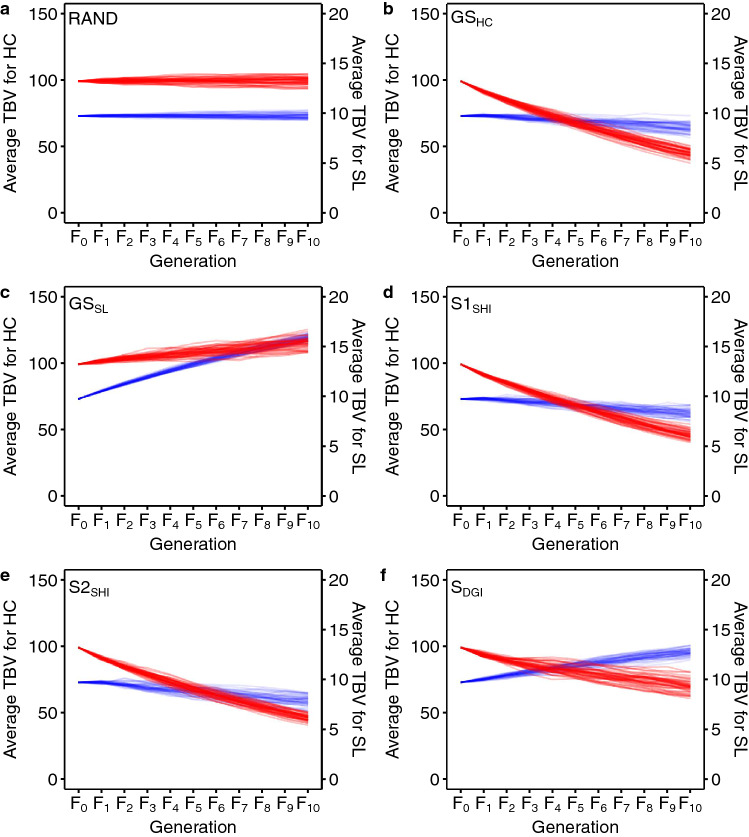
Genetic trends of average true breeding value (TBV) for *Heterobothrium okamotoi* count (HC, red lines) and standard length (SL, blue lines) of broodstock population in each generation (F_0_ to F_10_) among five different simulation scenarios with 50 replicates. (**a**) random mating (RAND), (**b**) GS on HC only (GS_HC_), (**c**) GS on SL only (GS_SL_), (**d**) Smith-Hazel index with the same economic weights S1_SHI_), (**e**) Smith-Hazel index with the different economic weights (S2_SHI_), and (**f**) desired gains index (S_DGI_).

The selection of one trait can have a negative impact on others when an unfavorable antagonistic correlation exists between traits. In this study, we tested the availability of LGSI methods for simultaneous improvements of HC and SL, assuming a genetic correlation estimated above (*r*_*g*_ = 0.228), using simulation studies. We simulated six scenarios each different in selection schemes, i.e. random mating (RAND), GS on HC only (GS_HC_), GS on SL only (GS_SL_), selection applying Smith-Hazel index^28,29^ (S1_SHI_ and S2_SHI_, different in economic weights) and desired gains index^31^ (S_DGI_), for 10 generations with 50 replications (Fig. 4). In short, RAND was based on random mating while GS_HC_ and GS_SL_ were based on GS on either of the traits. GEBV was estimated by GBLUP. In S1_SHI_, selection candidates were ranked based on the Smith-Hazel index. Since economic importance for each trait has not been evaluated in the tiger pufferfish aquaculture industry, we assume both traits have equal economic weights, which is *w* = [− 1, 1] for HC and SL for S1_SHI_ (HC is expected to decrease by selection). For S_DGI_, *d* was set as [− 3, 0.3] for HC and SL, so that SL can be improved preferentially while HC can be reduced by 30% after 10 generations (− 3 * 10/100 = − 30%). To compare the two selection index methods, we also ran an additional scenario (S2_SHI_) based on Smith-Hazel index, where the economic weight for each trait was set the same as the designed weights of S_DGI_ (*w* = [− 3, 0.3]). As expected, only S_DGI_ could improve the two traits simultaneously, where true breeding values (TBVs) of parasite load (HC) decreased while SL increased in each generation (Fig. 5).

## Discussion

In this study, we tested the possibility of GS for genetic improvements in heterobothriosis resistance of the tiger pufferfish from empirical data and conducted a simulation study to design a GS breeding strategy that could improve the resistance trait concurrent with growth-related traits. Overall, our results suggest GS for the parasite resistance trait is feasible (predictive ability = 0.248‒0.344) and breeding strategy incorporating the DGI method can simultaneously improve both HC and SL, even though an unfavorable antagonistic genetic correlation was suggested (*r*_*g*_ = 0.228).

With 6707 SNP makers, moderate estimated heritability of transformed HC (*h*_2_ = 0.308, SE = 0.123) and SL (*h*_2_ = 0.405, SE = 0.131) were obtained, indicating selective breeding for those traits is feasible. The estimated heritability was comparable to those estimated for resistance against sea lice in Atlantic salmon (*h*_2_ = 0.22 to 0.33 with 35 k SNPs)^22^ and bacterial cold water disease resistance (survival days) in farmed rainbow trout (*h*_2_ = 0.33 with 35 k SNPs)^9^. This suggested our small SNP panel could successfully capture the genetic variance for HC in the tiger pufferfish. In this study, we could not detect significant SNPs from GWAS. Even with the small SNP panel and small sample size, strong effect SNPs (the sex-determining SNP) could be detected in a cultured population of the tiger pufferfish^39^. Therefore, our GWAS result suggests the parasitic resistance is controlled by a large number of quantitative trait locus (QTL) with small or moderate effects, and marker-assisted selection is not feasible. This result is consistent with the previous QTL analysis using the interspecies hybrid system of pufferfishes^19^. Although the effect was not significant, genes neighboring the SNP with highest – log_10_(*p*) values on chromosome 9 (12,024,615 bp) deserve further investigation, because the genomic region including this site was reported to have a small QTL effect on host specificity of *H. okamotoi*^19^.

The predictive abilities for HC estimated under 12 models were moderate (0.248‒0.344), and within the range observed for other disease resistance traits examined in other fish species^21,23,24,42^. The predictive abilities of Bayesian hierarchical linear models (i.e. Bayes A, B, C, LASSO, and Ridge) were similar (0.303‒0.312) and scarcely higher than the GBLUP model (0.307 ± 0.018) for HC. This suggests that these linear models did not greatly differ regarding the predictive ability and the assumptions of the prior distribution of genetic effects have a limited impact on this trait. Bayes RKHS showed slightly better performance in HC compared to these linear models. For SVR-poly and SVR-rbf models, relatively low abilities for HC were observed, however, high abilities were found for SL. Since the default hyperparameters were used in the SVR models, hyperparameter tuning may aid achievement of better performance for HC as in the case of the previous study^43^. The architectures of FNN and multi-task FNN were tuned to achieve high predictive ability of GS for HC, however, the same architecture was applied to calculate the predictive ability of GS for SL. As expected, these models resulted in high predictive ability for HC but low for SL. This indicates that a deep learning model is task-specific and high accuracy can be obtained with careful optimization as described previously^44^. However, a great improvement in predictive ability was not achieved by FNN methods compared to GBLUP and Bayesian models even with the model complexity.

Our simulation study showed the availability of DGI for simultaneous genetic improvement in HC and SL even when the unfavorable antagonistic genetic correlation was assumed. The two scenarios incorporating the Smith-Hazel index showed the undesired consequences, where the average TBV for both SL and HC increased (smaller HC is favored). This happened because the breeding scheme only selected the individuals with the top LGSI values, but the high LGSI calculated by the Smith-Hazel index method does not guarantee the selected individuals are superior in both of the traits^45^, especially when target traits show a negative correlation. On the other hand, DGI, a variation of the selection index methods, allows selection with restrictions on multiple traits via the desired gains vector (*d*). In this study, the *d* vector was set with intending to reduce HC by 30% during 10 generations while maximizing SL. The desired gains vector (*d*) can be further optimized by comparing simulation scenarios with various *d* to achieve the self-defined breeding goal. Unfavorable genetic correlation between body size and disease resistance is commonly observed in aquaculture species, e.g. vibriosis in Atlantic cod^46^, bacterial cold water disease in rainbow trout^47^, and piscirickettsiosis in coho salmon^48^. Therefore, it is expected that DGI or the similar LGSI method can be widely applied for the simultaneous improvement of disease resistance trait and growth-related traits, which are the primary targets of most breeding programs.

In summary, the availability of GS for HC and SL was confirmed in this study. Moderate heritability for both traits suggests the genetic return from GS is high. GBLUP and Bayesian linear regression models showed similar predictive abilities for these traits. Although an unfavorable antagonistic genetic correlation was suggested between the two traits, the GS breeding strategy incorporating DGI can be a solution for the simultaneous genetic improvement.

## Methods

### Sample fish

The empirical experiments were performed in the Fisheries Laboratory, University of Tokyo (Hamamatsu, Shizuoka Prefecture, Japan). All samples (n = 240) were generated by a full-factorial mating among 10 wild males and 11 wild females, which were commercially caught from Wakasa Bay (Fukui Prefecture, Japan). For the mating, artificial fertilization was applied following the previous study^12^ with minor modification. In brief, females were anesthetized with 200 mg/l of 2-phenoxyethanol and then ripened by injection of 150 µg/kg of luteinizing hormone-releasing hormone (LHRH, Sigma-Aldrich, St. Louis, MP, USA). Gametes were stripped from each individual and fertilized per male–female pair (110 pairs in total). Fertilized eggs of each maternal half-sib family were mixed and kept in a hatching jar. After hatching, each maternal half-sib was kept in a holding tank for 1 month and then all families were mixed and cultured in a three-ton communal tank. Rearing and feeding conditions were set as previously described^10^. At 4 months age, 240 fish were randomly collected and subjected to the artificial challenge test.

### Artificial infection and phenotyping

Artificial infection was done following previous studies^12,49^. A day before the infection, fish were equally distributed into three identical one-ton experimental tanks (80 individuals/tank) supplied with *H. okamotoi*-free fresh seawater (UV treated and filtered). Meanwhile, eggs of *H. okamotoi* were collected from tanks containing infected fish and kept in a glass jar containing fresh seawater until infection. Hatching was induced by physical stimulation (shaking at 140 rpm for 10 min) and the density of oncomiracidia, the free-living larvae of *H. okamotoi*, in the suspensions was determined under the microscope just before the infection. At infection, the water depth of experimental tanks was adjusted to 15 cm, and approximately 4000 oncomiracidia was introduced into each tank. At 3 h post-exposure, fish were transferred into three, newly-setup one-ton holding tanks and reared for 32 days, when *H. okamotoi* reaches maturation and moves to the branchial cavity walls (BCW)^13^. At the 32-day mark, fish were euthanized, measured for SL and the BCWs dissected from both sides. For each fish, the caudal fin was clipped and kept in 600 µl TNE8U buffer^50^ (10 mM Tris–HCl (pH 7.5), 125 mM NaCl, 10 mM EDTA, 1% SDS, 8 M urea) at room temperature to extract genomic DNA for genotyping. Collected BCWs tissues were kept in 10% formalin until counting the number of parasites. The parasites attached to the whole BCWs were counted under a stereo microscope. The host resistance against *H. okamotoi* is assessed by parasite count on the entire BCWs (HC).

### Genotyping

Genomic DNA was extracted using a Gentra Puregene Tissue Kit (QIAGEN, Hilden, Germany) following the manufacture’s instruction and applied for AmpliSeq genotyping as previously described^39^. In short, 3187 genome-wide target regions were amplified by the first PCR with the custom AmpliSeq primer pools. After the adapter ligation and purification steps, PCR products were barcoded by a second PCR with 8-base index oligo sequences (Supplementary Table S1) for individual identification. The libraries of 240 individuals were pooled and sequenced on Illumina MiSeq System using the MiSeq reagent kit v2 (300 cycles) from both ends. The raw FASTQ reads were quality-trimmed using trimmomatic-0.36^51^ with the following parameters: *ILLUMINACLIP TruSeq3-PE-2. fa:2:30:10, LEADING:19, TRAILING:19, CROP:146, HEADCROP:5, SLIDINGWINDOW:30:20, AVGQUAL:20, and MINLEN:60*. Then, trimmed reads were mapped onto the target regions of the reference genome, FUGU5/fr3^52^ using BWA-MEM (vv0.7.12)^53^. Reads with mapping quality values (MAPQ) less than 10 were removed by samtools (v1.7-2)^54^. Genotype calling of each sample was done using GATK-4.1.6.0^55^ *HaplotypeCaller* with the following options: *–output-mode EMIT_ALL_CONFIDENT_SITES -ERC GVCF –stand-call-conf 30*. Obtained gVCF files were combined using GATK *CombineGVCFs* and then joint genotyping was performed using GATK *GenotypeGVCFs*. Variant filtering was done using vcftools (v0.1.5)^56^ with the following parameters: *–min-meanDP 15 –max-meanDP 500 –max-missing 0.7 –hwe 0.05 –minDP 10 –remove-indels*. The missing values of genotypes were imputed by LinkImputeR-1.2.1^40^. At first, SNPs which did not fulfill the maximum missingness per SNP and sample of 0.9 were filtered out, and then the missing genotypes were imputed. All samples were retained but 11 out of 6718 SNPs were discarded. Subsequently, the imputation accuracy was accessed with *numbermasked* option (set as 500). PLINK (v1.0.7)^57^ *–recodeA* option was used to generate the allele coding matrix from the imputed VCF files.

### Population structure

The population structure was analyzed with a nonlinear dimension reduction technique, *t*-distributed stochastic neighbor embedding (*t*-SNE)^41,58^. At first, *t*-SNE transforms the genomic data into conditional probabilities that represent pairwise similarity in the high-dimensional space. Then, transformed data were applied to a heavy-tailed Student’s *t*-distribution that measures pairwise similarities of corresponding samples in the low-dimensional embedding space. Finally, *t*-SNE minimized the sum of the Kullback–Leibler divergence between those two probability distributions (Kullback–Leibler divergence is the measure of the difference between two probability distributions). The *t*-SNE analysis was implemented in *sklearn.manifold.TSNE* class of Python/scikit-learn-0.20.3. The perplexity was set as 20 and default values were used for the other parameters.

### Heritability and genetic correlation

Heritability and genetic correlation were calculated by a multivariate linear mixed model as follows:

1 $$\begin{array}{c}{{\varvec{y}}}_{i}={{\varvec{X}}}_{i}{{\varvec{\beta}}}_{i}+{{\varvec{Z}}}_{i}{{\varvec{u}}}_{i}+{{\varvec{e}}}_{i},\end{array}$$

where ${{\varvec{y}}}_{i}$ is a vector of phenotypes for trait *i* (*i* = 1 for transformed HC and 2 for SL); ${{\varvec{X}}}_{i}$ and ${{\varvec{Z}}}_{i}$ are incidence matrices for fixed effects ${{\varvec{\beta}}}_{i}$ and random effects ${{\varvec{u}}}_{i}$, respectively. The model assumes the random effects (${\varvec{u}}$) follow a multivariate normal distribution as ${\varvec{u}} = {\left[{{\varvec{u}}}_{1}^{^{\prime}}{{\varvec{u}}}_{2}^{^{\prime}}\right]}^{^{\prime}} \sim MVN\left(0, {\varvec{G}}\otimes {\varvec{A}}\right)$ and the residuals (${\varvec{e}}$) follow ${\varvec{e}} = {\left[{{\varvec{e}}}_{1}^{^{\prime}}{{\varvec{e}}}_{2}^{^{\prime}}\right]}^{^{\prime}} \sim MVN\left(0, {\varvec{R}}\otimes {\varvec{I}}\right)$; where ${\varvec{G}}$ and ${\varvec{R}}$ are the variance–covariance matrices of random effects and residuals for the two traits, respectively; ${\varvec{A}}$ is the additive genetic relationship matrix constructed by *A.mat* function in R/sommer-4.0.1^59,60^ with the default settings; ${\varvec{I}}$ is the identity matrix; $\otimes$ means the operation of Kronecker product. The model was solved by *mmer* function in R/sommer-4.0.1 to solve the equations. The heritability (${h}_{i}^{2}$) was computed as:

2 $$\begin{array}{c}{h}_{i}^{2}=\frac{{\sigma }_{{g}_{i}}^{2}}{{\sigma }_{{g}_{i}}^{2}+{\sigma }_{{e}_{i}}^{2}},\end{array}$$

where ${\sigma }_{{g}_{i}}^{2}$ and ${\sigma }_{{e}_{i}}^{2}$ are the genetic variance and the residual variance for trait *i*, respectively. Then, the genetic correlation (${r}_{g}$) was computed as:

3 $$\begin{array}{c}{r}_{g}=\frac{{\sigma }_{{g}_{1},{g}_{2}}}{\sqrt{{{\sigma }_{{g}_{1}}^{2}\sigma }_{{g}_{2}}^{2}}},\end{array}$$

where ${\sigma }_{{g}_{1},{g}_{2}}$ is the genetic covariance between two traits.

The genetic correlation estimated by means of the multivariate model could be biased from the phenotypic correlation. Therefore, we further tested correlation between GEBV for each trait using GBLUP model. In the prediction model for HC, SL was included as the covariate to minimize non-genetic effects from SL. The prediction models are described as following:

4 $$\begin{array}{c}y=X\beta +Zu+\varepsilon ,\end{array}$$

where ${\varvec{y}}$ is a vector of phenotypes; ${\varvec{X}}$ is an incidence matrix for the fixed effect ${\varvec{\beta}}$ (for the prediction of HC, SL was added as a covariate); ${\varvec{Z}}$ is an identity matrix for the random effects ${\varvec{u}}$, which models the breeding values; ${\varvec{\varepsilon}}$ is a vector of residuals. The normality was assumed for random effects (${\varvec{u}}$) and residuals (${\varvec{\varepsilon}}$) as ${\varvec{u}} \sim N\left(0, {\varvec{K}}{\sigma }_{u}^{2}\right)$ and ${\varvec{\varepsilon}}\sim N\left(0,\boldsymbol{ }{\varvec{I}}{\sigma }_{\varepsilon }^{2}\right)$, respectively; where ${\varvec{K}}$ is a marker-based relationship matrix^61^; ${\varvec{I}}$ is an identity matrix; GEBVs were estimated by restricted maximum likelihood (REML) algorithm using *kin.blup* function in R/rrBLUP-4.6^62^.

### Genome-wide association study (GWAS)

To investigate the associated markers with transformed HC and SL, GWAS was performed based on the linear mixed model:

5 $$\begin{array}{c}y=X\beta +Zg+S\tau +\varepsilon ,\end{array}$$

where ${\varvec{y}}$ is the vector of the phenotypes; ${\varvec{\beta}}$ is a vector of fixed effects other than SNP effects; ${\varvec{g}}$ is the vector of random effects that models the polygene background; ${\varvec{\tau}}$ is a vector of fixed effects which represent the additive SNP effects; ${\varvec{X}}$, ${\varvec{Z}}$, and ${\varvec{S}}$ are incidence matrices relating to ${\varvec{\beta}}$, ${\varvec{g}}$, and ${\varvec{\tau}}$, respectively. ${\varvec{\varepsilon}}$ is a vector of normal residuals. ${\varvec{g}}$ and ${\varvec{\varepsilon}}$ follow the normal distributions as ${\varvec{g}} \sim N\left(0, {\varvec{K}}{\sigma }_{g}^{2}\right)$ and ${\varvec{\varepsilon}}\sim N\left(0,\boldsymbol{ }{\varvec{I}}{\sigma }_{\varepsilon }^{2}\right)$, respectively; where ${\varvec{K}}$ is the realized relationship matrix described above. A restricted maximum likelihood (REML) algorithm was performed to solve the linear mixed model using *GWAS* function of R/rrBLUP-4.6 with the parameter: n.PC = 10. The *p*-values were calculated for each SNP marker. The Bonferroni-corrected significant threshold was set as *α* = 7.454 × 10^–6^ (0.05 divided by the number of SNPs: 6707).

### Model comparison of genomic prediction

Predictive abilities were obtained under 12 regression models described below. The tenfold cross-validation scheme was applied for the ability calculation following the procedure described by Hosoya et al.^63^. Samples were randomly and equally divided into ten subsets: one for testing and the remaining for training. The phenotypic values of the test set were masked, and the regression model was trained using the training set. GEBVs of the test set were predicted and predictive ability was calculated as the correlation (Pearson's *r*) between GEBVs and observed values of the test set. This step was repeated with rotating the test sets among the ten subsets, and the average of Pearson's *r* was obtained. This cross-validation process was repeated 10 times to obtain the mean and the standard error of the mean (S.E.) for the predictive abilities. Transformed HC instead of the original phenotype was used in GBLUP and Bayesian models. To generate the consistent random state for sampling among 12 models, we fixed seeds for random sampling among the models.

### Genomic best linear unbiased prediction (GBLUP)

The same model as the Eq. (4) was used for GBLUP.

### Bayesian models

The models of Bayes A, B, C, Ridge, and LASSO^64,65^ are expressed as follows:

6 $$\begin{array}{c}y=\mu {1}_{n}+X\beta +\sum_{i=1}^{p}{{\varvec{z}}}_{i}{{\varvec{g}}}_{i}+\varepsilon ,\end{array}$$

where ${\varvec{y}}$**, X**, ${\varvec{\beta}}$ and ${\varvec{\varepsilon}}$ are same as GBLUP; $\mu$ is an intercept; ${1}_{n}$ is a vector of one; $p$ is the total number of genotypes for one individual; ${{\varvec{z}}}_{i}$ is a vector of genotypes at SNP *i*; ${{\varvec{g}}}_{i}$ is a vector of random effects that represent the genetic effects for SNP *i*. following a specific prior distribution. Bayes A assumes a scaled-*t* distribution for the prior while Bayes B assumes a mixture of gaussian distribution and a point mass at zero. The prior of Bayes C is a mixture of scaled-*t* distribution and a point of mass at zero. The prior of Bayes Ridge and Bayes LASSO is a normal distribution and a double exponential distribution, respectively. These models were solved using R/BGLR-1.0.8^66^ with nIter = 10,000 and burnIn = 2000.

### Bayesian reproducing kernel Hilbert spaces regression (Bayesian RKHS)

Bayesian RKHS is a Bayesian approach of semi-parametric regression^67^ structured as:

7 $$\begin{array}{c}y=\mu {1}_{n}+u+\varepsilon ,\end{array}$$

where each parameter is the same as the Bayesian models, while ${\varvec{u}}$ and ${\varvec{\varepsilon}}$ follow the normal distribution as ${\varvec{u}} \sim N(0,\hspace{0.5em}{\varvec{K}}{\sigma }_{u}^{2})$ and ${\varvec{\varepsilon}}\sim N(0,\hspace{0.5em}{\varvec{I}}{\sigma }_{\varepsilon }^{2})$, respectively; where ${\varvec{K}}$ is a reproducing kernel which approximates the marker-based relationship matrix and ${\varvec{I}}$ is an identity matrix. The model was solved using R/BGLR-1.0.8 with nIter = 10,000 and burnIn = 2000.

### Support vector machine regression (SVR)

The SVR method can be viewed as a convex optimization problem that finds a function from genotypes to phenotypes at most *ε*-deviation from all observed phenotypes while balancing the model complexity and prediction error^68,69^. The method of Lagrange multipliers is used to solve the optimization problem, and the derived approximate function follows:

8 $$\begin{array}{c}f\left({\varvec{x}}\right)=\sum_{i=1}^{N}\left({a}_{i}^{*}-{a}_{i}\right)k\left({\varvec{x}},{{\varvec{x}}}_{i}\right)+b,\end{array}$$

where the input ${\varvec{x}}$ is a vector of all genotypes for a single sample; $N$ is the sample size; ${a}_{i}^{*}$ and ${a}_{i}$ are Lagrange multipliers; $k\left({\varvec{x}},{{\varvec{x}}}_{i}\right)$ is a kernel function; ${{\varvec{x}}}_{i}$ is a vector of genotypes for individual $i$; $b$ is a residual. SVR-linear, SVR-poly, and SVR-rbf using linear, polynomial, and radial basis for kernel function, respectively. The SVR models were implemented by the *sklearn.svm.SVR* function in Python/scikit-learn-0.20.3^70^. The *gamma* parameter was set to ‘*auto*’ and the default setting was used for the other parameters.

### Neural networks

Feedforward neural networks (FNN), inspired by the biological neural network, can model genotype–phenotype regression^71^. Neural cells were modeled by non-linear functions (or activation function) and the network was mimicked by the chain structure. Our FNN had one input layer, two hidden layers, and a regression output. The number of input units was 6707, equivalent to the number of SNPs. The first hidden layer has 200 hidden units and the second 20. The rectified linear unit was used as an activation function in hidden layers. FNN was trained by minimizing the loss function, that is, the mean squared error in this case:

9 $$\begin{array}{c}MSE=\frac{1}{n}{\sum }_{i=1}^{n}{\left({y}_{i}-{\widehat{y}}_{i}\right)}^{2}\end{array}$$

where *n* is the sample size of the training group; ${y}_{i}$ and ${\widehat{y}}_{i}$ are observed value and the predicted value of individual $i$, respectively.

Multi-task deep neural network (Multi-task FNN) is based on an assumption where HC and SL share underlying genetic architecture to some extent. These models can improve the accuracy of estimation of the main output using the related auxiliary task as an inductive bias to the main task in reproducing kernel Hilbert space^72,73^. The model has one input layer, two hidden layers, one main regression output, and one auxiliary regression output. The first hidden layer has 200 units, which is a sharing layer for both tasks. Both outputs have separated second hidden layers that differ in the number of the hidden units (20 for the main trait and 100 units for the auxiliary trait). For the estimation of HC, the main regression output is for HC and auxiliary regression output is for SL. The model setting of the main regression and auxiliary regression output was reversed for SL estimation. The activation function and the loss function were the same as the FNN model described above. FNN and multi-task FNN were implemented in Python/keras-2.4.3 package^74^ with tensorflow-gpu-2.2.1 backend^75^. FNN used "Adam" optimizer, and multi-task FNN used "RMSprop" optimizer, both with the default parameters. Both models were trained by *model.fit* method in Python/keras with the parameters as epochs = 30, batch_size = 128, and others followed the default. Many combinations of model architecture, loss function, activate the function, and optimizer was arbitrarily chosen and tested to find the models here that have a high accuracy of GP for HC.

### Simulation study

To investigate the breeding strategy that can improve SL and HC simultaneously, six scenarios different in recurrent selection schemes were simulated for ten generations with 50 replicates using R/AlphaSimR-0.11.0 package^76^. The tested scenarios were named for the selection scheme applied: random mating (RAND), GS on HC only (GS_HC_), GS on SL only (GS_SL_), Smith-Hazel index (S1_SHI_ and S2_SHI_), and Desired gains index (S_DGI_). The workflow of the simulation study is illustrated in Fig. 4 and the details are described in the Supplementary material S2. LGSI forS1_SHI_, S2_SHI_ and S_DGI_ was constructed as:

10 $$\begin{array}{c}IGSI={{\varvec{b}}}^{^{\prime}}\widehat{{\varvec{y}}},\end{array}$$

where ${\varvec{b}}$ is a vector of index coefficients;$\widehat{{\varvec{y}}}$ is a vector of GEBVs. ${\varvec{b}}$ for S1_SHI_ and S2_SHI_ was computed as:

11 $$\begin{array}{c}b={{\varvec{P}}}^{-1}Aw,\end{array}$$

where ${\varvec{P}}$ and ${\varvec{A}}$ are phenotypic and genetic variance–covariance matrices, respectively; ${\varvec{w}}$ is the economic importance of both traits and set as [− 1, 1] assuming both traits have equal economic weights (HC is expected to be decreased by selection) for S1_SHI_. ${\varvec{P}}$ was obtained by *varP* function of R/AlphaSimR, and ${\varvec{A}}$ was obtained by *mmer* function of R/sommer-4.0.1 following the same procedure for the estimation of genetic correlation except that original HC value was used. On the other hand, ${\varvec{b}}$ for S_DGI_ was computed as:

12 $$\begin{array}{c}b={{\varvec{P}}}^{-1}A{({\varvec{A}}{\varvec{P}}}^{-1}A{)}^{-1}d,\end{array}$$

where ${\varvec{P}}$ and ${\varvec{A}}$ are the same as S_SHI_ while ${\varvec{d}}$ is a vector of desired gains. The combination of ${\varvec{d}}$ can be chosen arbitrarily depending on the breeding goal of the program. In this study, we set ${\varvec{d}}$ at [− 3, 0.3] for HC and SL so that SL can be improved preferentially while HC can be reduced by 30% after 10 generations. To compare different selection index methods, the vector of economic importance, [− 3, 0.3], which is the same as ${\varvec{d}}$ in S_DGI_ was assigned to ${\varvec{w}}$ in S2_SHI_.

### Ethics statement

All experiments done in this study were approved by and carried out in accordance with the IACUC (Institutional Animal Care and Use Committee) of the Graduate School of Agricultural and Life Sciences, University of Tokyo (P17-161). All methods were performed in accordance with the IACUC guidelines and regulations.

## Supplementary information

Supplementary Information.

## Data Availability

Amplicon sequence reads have been deposited in the DDBJ Sequence Read Archive (DRA Accession: DRA010341).

## References

[1] Gjedrem T, Robinson N, Rye M (2012). The importance of selective breeding in aquaculture to meet future demands for animal protein: A review. Aquaculture.

[2] Neira, R. Breeding in aquaculture species: genetic improvement programs in developing countries. In *The 9th World Congress on Genetics Applied to Livestock Production* 8 (2010).

[3] Rye, M., Gjerde, B. & Gjedrem, T. Genetic improvement programs for aquaculture species in developed countries. In *The 9th World Congress on Genetics Applied to Livestock Production* 8 (2010).

[4] Janssen K, Chavanne H, Berentsen P, Komen H (2017). Impact of selective breeding on European aquaculture. Aquaculture.

[5] Gjedrem T, Baranski M (2009). Selective Breeding in Aquaculture: An Introduction.

[6] Meuwissen THE, Hayes BJ, Goddard ME (2001). Prediction of total genetic value using genome-wide dense marker maps. Genetics.

[7] Robledo D, Palaiokostas C, Bargelloni L, Martínez P, Houston R (2018). Applications of genotyping by sequencing in aquaculture breeding and genetics. Rev. Aquac..

[8] Tsai HY (2015). Genome wide association and genomic prediction for growth traits in juvenile farmed Atlantic salmon using a high density SNP array. BMC Genomics.

[9] Vallejo RL (2017). Genomic selection models double the accuracy of predicted breeding values for bacterial cold water disease resistance compared to a traditional pedigree-based model in rainbow trout aquaculture. Genet. Sel. Evol..

[10] Hosoya S, Mizuno N, Kikuchi K, Kurokura H (2014). Rearing *Takifugu rubripes* larvae in communal tanks: Paternal genetic contribution to survivability. Fish. Sci..

[11] Yoshikawa S (2020). Precocious maturation in male tiger pufferfish *Takifugu rubripes*: Genetics and endocrinology. Fish. Sci..

[12] Kim DI (2019). Genetic variation in resistance of the tiger pufferfish *Takifugu rubripes* to a host-specific monogenean parasite *Heterobothrium okamotoi*. Fish. Sci..

[13] Ogawa K (2016). Heterobothriosis of cultured Japanese pufferfish *Takifugu rubripes*. Fish Pathol..

[14] Ogawa K (2002). Impacts of diclidophorid monogenean infections on fisheries in Japan. Int. J. Parasitol..

[15] Ogawa K, Inouye K (1997). Heterobothium infection of cultured tiger puffer, *Takifugu rubripes*—A field observation. Fish Pathol..

[16] Shirakashi S, Nakatsuka S, Udagawa A, Ogawa K (2010). Oncomiracidial Behavior of *Heterobothrium okamotoi* (Monogenea: Diclidophoridae). Fish Pathol..

[17] Igarashi K (2017). Mucosal IgM antibody with d-Mannose affinity in fugu *Takifugu rubripes* is utilized by a Monogenean parasite *Heterobothrium okamotoi* for host recognition. J. Immunol..

[18] Matsui S (2020). d-mannose-specific immunoglobulin M in grass puffer (*Takifugu niphobles*), a nonhost fish of a monogenean ectoparasite *Heterobothrium okamotoi*, can act as a trigger for its parasitism. J. Parasitol..

[19] Hosoya S (2013). Genomic regions of pufferfishes responsible for host specificity of a monogenean parasite, *Heterobothrium okamotoi*. Int. J. Parasitol..

[20] Moen T (2015). Epithelial cadherin determines resistance to infectious pancreatic necrosis virus in Atlantic salmon. Genetics.

[21] Robledo D, Matika O, Hamilton A, Houston RD (2018). Genome-wide association and genomic selection for resistance to amoebic gill disease in Atlantic salmon. G3 Genes Genomes Genet..

[22] Tsai HY (2016). Genomic prediction of host resistance to sea lice in farmed Atlantic salmon populations. Genet. Sel. Evol..

[23] Palaiokostas C (2018). Genome-wide association and genomic prediction of resistance to viral nervous necrosis in European sea bass (*Dicentrarchus labrax*) using RAD sequencing. Genet. Sel. Evol..

[24] Palaiokostas C, Ferraresso S, Franch R, Houston RD, Bargelloni L (2016). Genetics of resistance to photobacteriosis in gilthead sea bream (*Sparus aurata*) using 2b-RAD sequencing. G3..

[25] Ødegård J, Baranski M, Gjerde B, Gjedrem T (2011). Methodology for genetic evaluation of disease resistance in aquaculture species: Challenges and future prospects. Aquac. Res..

[26] Lynch M, Walsh B (1998). Genetics and Analysis of Quantitative Traits.

[27] Gjerde B, Ødegård J, Thorland I (2011). Estimates of genetic variation in the susceptibility of Atlantic salmon (*Salmo salar*) to the salmon louse *Lepeophtheirus salmonis*. Aquaculture.

[28] Smith HF (1936). A discriminant function for plant selection. Ann. Eugen..

[29] Hazel LN (1943). The genetic basis for constructing selection indexes. Genetics.

[30] Cerón-Rojas JJ, Crossa J (2018). Linear Selection Indices in Modern Plant Breeding.

[31] Itoh Y, Yamada Y (1987). Comparisons of selection indices achieving predetermined proportional gains. Genet. Sel. Evol..

[32] Ceron-Rojas JJ (2015). A genomic selection index applied to simulated and real data. G3 Genes Genomes Genet..

[33] Togashi K, Lin CY, Yamazaki T (2011). The efficiency of genome-wide selection for genetic improvement of net merit. J. Anim. Sci..

[34] Daetwyler HD, Pong-Wong R, Villanueva B, Woolliams JA (2010). The impact of genetic architecture on genome-wide evaluation methods. Genetics.

[35] Guo Z (2014). The impact of population structure on genomic prediction in stratified populations. Theor. Appl. Genet..

[36] Solberg TR, Sonesson AK, Woolliams JA, Meuwissen THE (2008). Genomic selection using different marker types and densities. J. Anim. Sci..

[37] Daetwyler HD, Calus MPL, Pong-Wong R, de los Campos G, Hickey JM (2013). Genomic prediction in animals and plants: Simulation of data, validation, reporting, and benchmarking. Genetics.

[38] Wang Q (2019). Evaluation on the genomic selection in *Litopenaeus vannamei* for the resistance against Vibrio parahaemolyticus. Aquaculture.

[39] Sato M (2019). A highly flexible and repeatable genotyping method for aquaculture studies based on target amplicon sequencing using next-generation sequencing technology. Sci. Rep..

[40] Money D, Migicovsky Z, Gardner K, Myles S (2017). LinkImputeR: User-guided genotype calling and imputation for non-model organisms. BMC Genomics.

[41] Van Der Maaten LJP, Hinton GE (2008). Visualizing high-dimensional data using t-sne. J. Mach. Learn. Res..

[42] Odegård J (2014). Genomic prediction in an admixed population of Atlantic salmon (*Salmo salar*). Front. Genet..

[43] Azodi CB (2019). Benchmarking parametric and machine learning models for genomic prediction of complex traits. G3 Genes Genomes Genet..

[44] Pérez-Enciso M, Zingaretti LM (2019). A guide for using deep learning for complex trait genomic prediction. Genes.

[45] Kempthorne O, Nordskog AW (1959). Restricted selection indices. Biometrics.

[46] Bangera R, Ødegård J, Præbel AK, Mortensen A, Nielsen HM (2011). Genetic correlations between growth rate and resistance to vibriosis and viral nervous necrosis in Atlantic cod (*Gadus morhua* L). Aquaculture.

[47] Evenhuis JP, Leeds TD, Marancik DP, Lapatra SE, Wiens GD (2015). Rainbow trout (*Oncorhynchus mykiss*) resistance to columnaris disease is heritable and favorably correlated with bacterial cold water disease resistance. J. Anim. Sci..

[48] Yáñez JM (2016). Negative genetic correlation between resistance against *Piscirickettsia salmonis* and harvest weight in coho salmon (*Oncorhynchus kisutch*). Aquaculture.

[49] Chigasaki M, Nakane M, Ogawa K, Wakabayashi H (2000). Standardized method for experimental infection of tiger puffer Takifugu rubripes with oncomiracidia of *Heterobothrium okamotoi* (Monogenea: Diclidophoridae) with some data on the oncomiracidial biology. Fish Pathol..

[50] Asahida T, Kobayashi T, Saitoh K, Nakayama I (1996). Tissue preservation and total DNA extraction from fish stored at ambient temperature using buffers containing high concentration of urea. Fish. Sci..

[51] Bolger AM, Lohse M, Usadel B (2014). Trimmomatic: A flexible trimmer for Illumina sequence data. Bioinformatics.

[52] Kai W (2011). Integration of the genetic map and genome assembly of fugu facilitates insights into distinct features of genome evolution in teleosts and mammals. Genome Biol. Evol..

[53] Li, H. Aligning sequence reads, clone sequences and assembly contigs with BWA-MEM. arXiv preprint, arXiv:1303.3997. (2013).

[54] Li H (2009). The sequence alignment/map format and SAMtools. Bioinformatics.

[55] Poplin, R. *et al.* Scaling accurate genetic variant discovery to tens of thousands of samples. *bioRxiv* 201178. Doi: 10.1101/201178 (2017)

[56] Danecek P (2011). The variant call format and VCFtools. Bioinformatics.

[57] Purcell S (2007). PLINK: A tool set for whole-genome association and population-based linkage analyses. Am. J. Hum. Genet..

[58] Amir EAD (2013). viSNE enables visualization of high dimensional single-cell data and reveals phenotypic heterogeneity of leukemia. Nat. Biotechnol..

[59] Covarrubias-Pazaran G (2018). Software update: Moving the R package sommer to multivariate mixed models for genome-assisted prediction. bioRxiv..

[60] Covarrubias-Pazaran G (2016). Genome-assisted prediction of quantitative traits using the R package sommer. PLoS ONE.

[61] Endelman JB, Jannink J-L (2012). Shrinkage estimation of the realized relationship matrix. G3 Genes Genomes Genet..

[62] Endelman JB (2011). Ridge regression and other kernels for genomic selection with R package rrBLUP. Plant Genome J..

[63] Hosoya S (2018). Assessment of genetic diversity in Coho salmon (*Oncorhynchus kisutch*) populations with no family records using ddRAD-seq. BMC Res. Notes.

[64] Habier D, Fernando RL, Kizilkaya K, Garrick DJ (2011). Extension of the bayesian alphabet for genomic selection. BMC Bioinform..

[65] Park T, Casella G (2008). The Bayesian Lasso. J. Am. Stat. Assoc..

[66] Pérez P, De Los Campos G (2014). Genome-wide regression and prediction with the BGLR statistical package. Genetics.

[67] De Los Campos G, Gianola D, Rosa GJM, Weigel KA, Crossa J (2010). Semi-parametric genomic-enabled prediction of genetic values using reproducing kernel Hilbert spaces methods. Genet. Res..

[68] Vapnik, V. N. *The Nature of Statistical Learning Theory*. 10.1007/978-1-4757-2440-0 (1995)

[69] Awad, M., Khanna, R., Awad, M. & Khanna, R. Support vector regression. In *Efficient Learning Machines* 67–80 (Apress, New York, 2015).

[70] Pedregosa F (2011). Scikit-learn: Machine learning in Python. J. Mach. Learn. Res..

[71] Gianola D, Okut H, Weigel KA, Rosa GJ (2011). Predicting complex quantitative traits with Bayesian neural networks: A case study with Jersey cows and wheat. BMC Genet..

[72] Caruana R (1997). Multitask learning. Mach. Learn..

[73] Widmer, C. & Rätsch, G. Multitask learning in computational biology. In *The 2011 International Conference on Unsupervised and Transfer Learning Workshop*, Vol. **27**, 207–216 (2011).

[74] Chollet, F. Keras: Deep learning library for theano and tensorflow. *GitHub Repositiry*. https://github.com/fchollet/keras (2015).

[75] Abadi, M. *et al.* TensorFlow: Large-scale machine learning on heterogeneous distributed systems. (2016).

[76] Gaynor RC, Gorjanc G, Hickey JM (2020). AlphaSimR: An R-package for breeding program simulations. bioRxiv.

