# Supplementary material for: Genomic selection for heterobothriosis resistance concurrent with body size in the tiger pufferfish, Takifugu rubripes
Source: Sci Rep. 2020 Nov 17;10:19976. doi: 10.1038/s41598-020-77069-z (PMC7672106; doi:10.1038/s41598-020-77069-z)
Supplement: Supplementary file 1 — Supplementary Information. [file 41598_2020_77069_MOESM1_ESM.docx]

Article title: Genomic selection for heterobothriosis resistance concurrent with body size in the tiger pufferfish, *Takifugu rubripes*

Authors: Zijie Lin^1^, Sho Hosoya^1, *^, Mana Sato^1^, Naoki Mizuno^1^, Yuki Kobayashi^2^, Takuya Itou^2^, Kiyoshi Kikuchi^1^

^1^Fisheries Laboratory, University of Tokyo, Hamamatsu, 431-0214, Japan

^2^Veterinary Research Center, Nihon University, Kanagawa, 252-0880, Japan

*Corresponding author: Sho Hosoya

e-mail: ahosoya@mail.ecc.u-tokyo.ac.jp

TEL: +81-53-592-2821; FAX: +81-53-592-282

**Supplementary material S1.** Phenotype information and index sequence for Ampliseq of each individual

**Supplementary material S2.** Workflow of simulation

**Supplementary material S1. Phenotype information and index sequence for Ampliseq of each individual**

| Sample ID | SL (standard length) | HC (Heterobothriosis count) | Forward_index | Reverse_index |
| --- | --- | --- | --- | --- |
| 1 | 7.9 | 27 | ATTACTCG | TATAGCCT |
| 2 | 8.4 | 17 | ATTACTCG | ATAGAGGC |
| 3 | 9.8 | 18 | ATTACTCG | CCTATCCT |
| 4 | 11.2 | 4 | ATTACTCG | GGCTCTGA |
| 5 | 9 | 17 | ATTACTCG | AGGCGAAG |
| 6 | 10.1 | 10 | ATTACTCG | TAATCTTA |
| 7 | 10 | 18 | ATTACTCG | CAGGACGT |
| 8 | 9.5 | 13 | ATTACTCG | GTACTGAC |
| 9 | 8.3 | 9 | TCCGGAGA | TATAGCCT |
| 10 | 8.8 | 5 | TCCGGAGA | ATAGAGGC |
| 11 | 9.3 | 16 | TCCGGAGA | CCTATCCT |
| 12 | 9.2 | 19 | TCCGGAGA | GGCTCTGA |
| 13 | 10.2 | 9 | TCCGGAGA | AGGCGAAG |
| 14 | 8.8 | 14 | TCCGGAGA | TAATCTTA |
| 15 | 10.2 | 17 | TCCGGAGA | CAGGACGT |
| 16 | 9.1 | 8 | TCCGGAGA | GTACTGAC |
| 17 | 9.4 | 6 | CGCTCATT | TATAGCCT |
| 18 | 9 | 25 | CGCTCATT | ATAGAGGC |
| 19 | 10.9 | 11 | CGCTCATT | CCTATCCT |
| 20 | 8.8 | 4 | CGCTCATT | GGCTCTGA |
| 21 | 10.6 | 13 | CGCTCATT | AGGCGAAG |
| 22 | 10.5 | 20 | CGCTCATT | TAATCTTA |
| 23 | 9.2 | 11 | CGCTCATT | CAGGACGT |
| 24 | 9.4 | 7 | CGCTCATT | GTACTGAC |
| 25 | 11.3 | 18 | GAGATTCC | TATAGCCT |
| 26 | 10.9 | 29 | GAGATTCC | ATAGAGGC |
| 27 | 10.2 | 11 | GAGATTCC | CCTATCCT |
| 28 | 9.5 | 11 | GAGATTCC | GGCTCTGA |
| 29 | 10.2 | 9 | GAGATTCC | AGGCGAAG |
| 30 | 7.9 | 10 | GAGATTCC | TAATCTTA |
| 31 | 11 | 15 | GAGATTCC | CAGGACGT |
| 32 | 10.2 | 15 | GAGATTCC | GTACTGAC |
| 33 | 10.3 | 17 | ATTCAGAA | TATAGCCT |
| 34 | 9.8 | 12 | ATTCAGAA | ATAGAGGC |
| 35 | 9.9 | 15 | ATTCAGAA | CCTATCCT |
| 36 | 9.7 | 33 | ATTCAGAA | GGCTCTGA |
| 37 | 11.7 | 25 | ATTCAGAA | AGGCGAAG |
| 38 | 10.2 | 14 | ATTCAGAA | TAATCTTA |
| 39 | 10.1 | 13 | ATTCAGAA | CAGGACGT |
| 40 | 10.2 | 12 | ATTCAGAA | GTACTGAC |
| 41 | 10.7 | 24 | GAATTCGT | TATAGCCT |
| 42 | 10.5 | 20 | GAATTCGT | ATAGAGGC |
| 43 | 11.6 | 22 | GAATTCGT | CCTATCCT |
| 44 | 11.5 | 49 | GAATTCGT | GGCTCTGA |
| 45 | 9.7 | 4 | GAATTCGT | AGGCGAAG |
| 46 | 9.5 | 16 | GAATTCGT | TAATCTTA |
| 47 | 9.4 | 15 | GAATTCGT | CAGGACGT |
| 48 | 10.1 | 10 | GAATTCGT | GTACTGAC |
| 49 | 10.3 | 23 | CTGAAGCT | TATAGCCT |
| 50 | 10 | 31 | CTGAAGCT | ATAGAGGC |
| 51 | 10.5 | 21 | CTGAAGCT | CCTATCCT |
| 52 | 9.7 | 5 | CTGAAGCT | GGCTCTGA |
| 53 | 9.6 | 6 | CTGAAGCT | AGGCGAAG |
| 54 | 9.5 | 10 | CTGAAGCT | TAATCTTA |
| 55 | 10.2 | 18 | CTGAAGCT | CAGGACGT |
| 56 | 8.8 | 15 | CTGAAGCT | GTACTGAC |
| 57 | 10.8 | 13 | TAATGCGC | TATAGCCT |
| 58 | 10.7 | 8 | TAATGCGC | ATAGAGGC |
| 59 | 9 | 2 | TAATGCGC | CCTATCCT |
| 60 | 9.5 | 13 | TAATGCGC | GGCTCTGA |
| 61 | 8.8 | 10 | TAATGCGC | AGGCGAAG |
| 62 | 10.4 | 7 | TAATGCGC | TAATCTTA |
| 63 | 8.8 | 8 | TAATGCGC | CAGGACGT |
| 64 | 9.3 | 2 | TAATGCGC | GTACTGAC |
| 65 | 8.8 | 0 | CGGCTATG | TATAGCCT |
| 66 | 9.6 | 23 | CGGCTATG | ATAGAGGC |
| 67 | 10.7 | 6 | CGGCTATG | CCTATCCT |
| 68 | 9.8 | 15 | CGGCTATG | GGCTCTGA |
| 69 | 10.8 | 12 | CGGCTATG | AGGCGAAG |
| 70 | 10.4 | 18 | CGGCTATG | TAATCTTA |
| 71 | 9.4 | 1 | CGGCTATG | CAGGACGT |
| 72 | 9.2 | 14 | CGGCTATG | GTACTGAC |
| 73 | 9.9 | 11 | TCCGCGAA | TATAGCCT |
| 74 | 8.8 | 20 | TCCGCGAA | ATAGAGGC |
| 75 | 8.7 | 18 | TCCGCGAA | CCTATCCT |
| 76 | 8.4 | 12 | TCCGCGAA | GGCTCTGA |
| 77 | 8.8 | 38 | TCCGCGAA | AGGCGAAG |
| 78 | 8.7 | 31 | TCCGCGAA | TAATCTTA |
| 79 | 10.2 | 20 | TCCGCGAA | CAGGACGT |
| 80 | 9.8 | 19 | TCCGCGAA | GTACTGAC |
| 81 | 11.1 | 35 | TCTCGCGC | TATAGCCT |
| 82 | 10.2 | 0 | TCTCGCGC | ATAGAGGC |
| 83 | 10.7 | 17 | TCTCGCGC | CCTATCCT |
| 84 | 10.1 | 18 | TCTCGCGC | GGCTCTGA |
| 85 | 10.6 | 11 | TCTCGCGC | AGGCGAAG |
| 86 | 10.4 | 35 | TCTCGCGC | TAATCTTA |
| 87 | 9.7 | 14 | TCTCGCGC | CAGGACGT |
| 88 | 9.3 | 10 | TCTCGCGC | GTACTGAC |
| 89 | 9 | 11 | AGCGATAG | TATAGCCT |
| 90 | 9.6 | 15 | AGCGATAG | ATAGAGGC |
| 91 | 10.7 | 22 | AGCGATAG | CCTATCCT |
| 92 | 10.5 | 16 | AGCGATAG | GGCTCTGA |
| 93 | 9.1 | 15 | AGCGATAG | AGGCGAAG |
| 94 | 10.1 | 24 | AGCGATAG | TAATCTTA |
| 95 | 8.9 | 9 | AGCGATAG | CAGGACGT |
| 96 | 10.5 | 0 | AGCGATAG | GTACTGAC |
| 97 | 9.4 | 24 | ATTACTCG | CCTGCGAA |
| 98 | 9.9 | 7 | ATTACTCG | TGCAGAGA |
| 99 | 9.5 | 27 | ATTACTCG | ACCTAGGA |
| 100 | 11 | 31 | ATTACTCG | TTGATCCA |
| 101 | 9.7 | 4 | ATTACTCG | ATCTTGCA |
| 102 | 9.4 | 14 | ATTACTCG | TCTCCATA |
| 103 | 11.3 | 27 | ATTACTCG | CATCGAGA |
| 104 | 9.8 | 15 | ATTACTCG | TTCGAGCA |
| 105 | 9.5 | 7 | TCCGGAGA | CCTGCGAA |
| 106 | 9.3 | 11 | TCCGGAGA | TGCAGAGA |
| 107 | 10.1 | 18 | TCCGGAGA | ACCTAGGA |
| 108 | 9.1 | 15 | TCCGGAGA | TTGATCCA |
| 109 | 8.5 | 23 | TCCGGAGA | ATCTTGCA |
| 110 | 9.7 | 30 | TCCGGAGA | TCTCCATA |
| 111 | 9.6 | 7 | TCCGGAGA | CATCGAGA |
| 112 | 10.4 | 18 | TCCGGAGA | TTCGAGCA |
| 113 | 8.9 | 42 | CGCTCATT | CCTGCGAA |
| 114 | 10.1 | 9 | CGCTCATT | TGCAGAGA |
| 115 | 9.7 | 12 | CGCTCATT | ACCTAGGA |
| 116 | 10.2 | 9 | CGCTCATT | TTGATCCA |
| 117 | 9.8 | 7 | CGCTCATT | ATCTTGCA |
| 118 | 9.6 | 34 | CGCTCATT | TCTCCATA |
| 119 | 8.8 | 11 | CGCTCATT | CATCGAGA |
| 120 | 9.5 | 15 | CGCTCATT | TTCGAGCA |
| 121 | 10.3 | 12 | GAGATTCC | CCTGCGAA |
| 122 | 10.1 | 20 | GAGATTCC | TGCAGAGA |
| 123 | 9.6 | 26 | GAGATTCC | ACCTAGGA |
| 124 | 9.4 | 21 | GAGATTCC | TTGATCCA |
| 125 | 8.7 | 14 | GAGATTCC | ATCTTGCA |
| 126 | 9.5 | 21 | GAGATTCC | TCTCCATA |
| 127 | 9.9 | 19 | GAGATTCC | CATCGAGA |
| 128 | 9.7 | 7 | GAGATTCC | TTCGAGCA |
| 129 | 9 | 27 | ATTCAGAA | CCTGCGAA |
| 130 | 10.4 | 35 | ATTCAGAA | TGCAGAGA |
| 131 | 9.8 | 37 | ATTCAGAA | ACCTAGGA |
| 132 | 10.2 | 17 | ATTCAGAA | TTGATCCA |
| 133 | 8.9 | 14 | ATTCAGAA | ATCTTGCA |
| 134 | 9.6 | 15 | ATTCAGAA | TCTCCATA |
| 135 | 9.8 | 42 | ATTCAGAA | CATCGAGA |
| 136 | 10.2 | 37 | ATTCAGAA | TTCGAGCA |
| 137 | 9.9 | 24 | GAATTCGT | CCTGCGAA |
| 138 | 8.9 | 21 | GAATTCGT | TGCAGAGA |
| 139 | 9.8 | 36 | GAATTCGT | ACCTAGGA |
| 140 | 9 | 16 | GAATTCGT | TTGATCCA |
| 141 | 9.2 | 22 | GAATTCGT | ATCTTGCA |
| 142 | 9.3 | 8 | GAATTCGT | TCTCCATA |
| 143 | 10.9 | 31 | GAATTCGT | CATCGAGA |
| 144 | 11.1 | 7 | GAATTCGT | TTCGAGCA |
| 145 | 10.4 | 27 | CTGAAGCT | CCTGCGAA |
| 146 | 10.2 | 16 | CTGAAGCT | TGCAGAGA |
| 147 | 8.6 | 6 | CTGAAGCT | ACCTAGGA |
| 148 | 9.4 | 16 | CTGAAGCT | TTGATCCA |
| 149 | 10 | 13 | CTGAAGCT | ATCTTGCA |
| 150 | 9.4 | 9 | CTGAAGCT | TCTCCATA |
| 151 | 8.9 | 9 | CTGAAGCT | CATCGAGA |
| 152 | 10.5 | 20 | CTGAAGCT | TTCGAGCA |
| 153 | 8.4 | 3 | TAATGCGC | CCTGCGAA |
| 154 | 11.4 | 8 | TAATGCGC | TGCAGAGA |
| 155 | 10.4 | 16 | TAATGCGC | ACCTAGGA |
| 156 | 9.1 | 19 | TAATGCGC | TTGATCCA |
| 157 | 8.6 | 8 | TAATGCGC | ATCTTGCA |
| 158 | 9.1 | 8 | TAATGCGC | TCTCCATA |
| 159 | 8.5 | 6 | TAATGCGC | CATCGAGA |
| 160 | 9.8 | 21 | TAATGCGC | TTCGAGCA |
| 161 | 10.2 | 14 | CGGCTATG | CCTGCGAA |
| 162 | 9.6 | 14 | CGGCTATG | TGCAGAGA |
| 163 | 8.7 | 2 | CGGCTATG | ACCTAGGA |
| 164 | 9.8 | 21 | CGGCTATG | TTGATCCA |
| 165 | 10.5 | 32 | CGGCTATG | ATCTTGCA |
| 166 | 10.9 | 19 | CGGCTATG | TCTCCATA |
| 167 | 8.8 | 7 | CGGCTATG | CATCGAGA |
| 168 | 10.7 | 18 | CGGCTATG | TTCGAGCA |
| 169 | 10.2 | 0 | TCCGCGAA | CCTGCGAA |
| 170 | 11.1 | 18 | TCCGCGAA | TGCAGAGA |
| 171 | 8.7 | 7 | TCCGCGAA | ACCTAGGA |
| 172 | 9.7 | 18 | TCCGCGAA | TTGATCCA |
| 173 | 10.5 | 11 | TCCGCGAA | ATCTTGCA |
| 174 | 11.5 | 6 | TCCGCGAA | TCTCCATA |
| 175 | 10.8 | 4 | TCCGCGAA | CATCGAGA |
| 176 | 10.2 | 2 | TCCGCGAA | TTCGAGCA |
| 177 | 9.8 | 7 | TCTCGCGC | CCTGCGAA |
| 178 | 9.6 | 9 | TCTCGCGC | TGCAGAGA |
| 179 | 10.7 | 4 | TCTCGCGC | ACCTAGGA |
| 180 | 10.1 | 14 | TCTCGCGC | TTGATCCA |
| 181 | 10.4 | 2 | TCTCGCGC | ATCTTGCA |
| 182 | 9.8 | 21 | TCTCGCGC | TCTCCATA |
| 183 | 9.9 | 33 | TCTCGCGC | CATCGAGA |
| 184 | 10.3 | 2 | TCTCGCGC | TTCGAGCA |
| 185 | 10.3 | 22 | AGCGATAG | CCTGCGAA |
| 186 | 10.5 | 15 | AGCGATAG | TGCAGAGA |
| 187 | 10.6 | 37 | AGCGATAG | ACCTAGGA |
| 188 | 10.1 | 6 | AGCGATAG | TTGATCCA |
| 189 | 10.2 | 13 | AGCGATAG | ATCTTGCA |
| 190 | 9.8 | 22 | AGCGATAG | TCTCCATA |
| 191 | 10.3 | 19 | AGCGATAG | CATCGAGA |
| 192 | 10.2 | 24 | AGCGATAG | TTCGAGCA |
| 193 | 10.9 | 13 | CCGGTACA | TATAGCCT |
| 194 | 9.4 | 30 | CCGGTACA | ATAGAGGC |
| 195 | 10.5 | 20 | CCGGTACA | CCTATCCT |
| 196 | 9.3 | 8 | CCGGTACA | GGCTCTGA |
| 197 | 10.4 | 11 | CCGGTACA | AGGCGAAG |
| 198 | 9.3 | 3 | CCGGTACA | TAATCTTA |
| 199 | 11.4 | 11 | CCGGTACA | CAGGACGT |
| 200 | 9.3 | 21 | CCGGTACA | GTACTGAC |
| 201 | 9.2 | 14 | AACTCCGA | TATAGCCT |
| 202 | 9.5 | 16 | AACTCCGA | ATAGAGGC |
| 203 | 10.4 | 32 | AACTCCGA | CCTATCCT |
| 204 | 11.1 | 17 | AACTCCGA | GGCTCTGA |
| 205 | 8.8 | 8 | AACTCCGA | AGGCGAAG |
| 206 | 9.4 | 3 | AACTCCGA | TAATCTTA |
| 207 | 9.1 | 12 | AACTCCGA | CAGGACGT |
| 208 | 8.9 | 22 | AACTCCGA | GTACTGAC |
| 209 | 10.1 | 11 | TTGAAGTA | TATAGCCT |
| 210 | 9.7 | 17 | TTGAAGTA | ATAGAGGC |
| 211 | 8.4 | 13 | TTGAAGTA | CCTATCCT |
| 212 | 10.4 | 23 | TTGAAGTA | GGCTCTGA |
| 213 | 10.2 | 31 | TTGAAGTA | AGGCGAAG |
| 214 | 9.5 | 14 | TTGAAGTA | TAATCTTA |
| 215 | 9.8 | 10 | TTGAAGTA | CAGGACGT |
| 216 | 10.7 | 26 | TTGAAGTA | GTACTGAC |
| 217 | 9.9 | 17 | ACTATCAA | TATAGCCT |
| 218 | 10.6 | 21 | ACTATCAA | ATAGAGGC |
| 219 | 9.5 | 18 | ACTATCAA | CCTATCCT |
| 220 | 10.3 | 6 | ACTATCAA | GGCTCTGA |
| 221 | 9.5 | 6 | ACTATCAA | AGGCGAAG |
| 222 | 9.3 | 21 | ACTATCAA | TAATCTTA |
| 223 | 10.4 | 6 | ACTATCAA | CAGGACGT |
| 224 | 10.6 | 14 | ACTATCAA | GTACTGAC |
| 225 | 10.6 | 10 | CGACCTGA | TATAGCCT |
| 226 | 9.1 | 3 | CGACCTGA | ATAGAGGC |
| 227 | 10.4 | 10 | CGACCTGA | CCTATCCT |
| 228 | 10.3 | 11 | CGACCTGA | GGCTCTGA |
| 229 | 9.5 | 23 | CGACCTGA | AGGCGAAG |
| 230 | 10.4 | 18 | CGACCTGA | TAATCTTA |
| 231 | 11.4 | 20 | CGACCTGA | CAGGACGT |
| 232 | 11.3 | 31 | CGACCTGA | GTACTGAC |
| 233 | 11.2 | 29 | AGGTACCA | TATAGCCT |
| 234 | 10.3 | 8 | AGGTACCA | ATAGAGGC |
| 235 | 9.7 | 24 | AGGTACCA | CCTATCCT |
| 236 | 10.2 | 13 | AGGTACCA | GGCTCTGA |
| 237 | 9.6 | 17 | AGGTACCA | AGGCGAAG |
| 238 | 7.7 | 17 | AGGTACCA | TAATCTTA |
| 239 | 8.4 | 35 | AGGTACCA | CAGGACGT |
| 240 | 8.7 | 8 | AGGTACCA | GTACTGAC |

**Supplementary material S2: Workflow of simulation**

**Workflow of simulation**

The simulated population was generated by *runMaCS2* function in R/AlphaSimR package^1^. First, all scenarios began with a founder population of 10,000 individuals was simulated assuming: the effective population size of 1,000, mutation rate of 2.5 x 10^-8^_,_ no inbreeding in founder individuals. The ploidy (*n* = 2), the number of chromosomes (*n* = 22), and genetic and physical size of each chromosome (Morgans and base pairs, respectively) were set according to the reference genome sequence integrated with the genetic map of the tiger pufferfish, FUGU5/fr3^2^. The relative ratio of recombination in females compared to males was set as 1.82 according to FUGU5/fr3. The phenotypic mean of SL was set in accordance with the phenotyping result and that of HC was set as 100 to avoid minus values of phenotypes after genetic improvement. Phenotypic variance, genetic variance, heritability, and genetic correlation were simulated referring to the analysis result using empirical data obtained in this study. The gender of each individual was randomly assigned. For each trait, 500 QTLs were placed per chromosome. The number of SNP markers per chromosome was equal to that detected in the Ampliseq custom panel (*n* = 6,707 in total) and randomly placed over each chromosome to form an SNP chip *in silico*. To initiate the GS breeding program, 20 sires and 20 dams were randomly sampled from the founder population to perform a full-factorial mating, and then e­­ach parent pair generates 20 progenies and in total 8,000 progenies were produced. From the progeny pool, 2,000 fish were randomly picked up as the broodstock population (F_0_). The relatively small number of parents were used in this simulation study compared with the practical breeding programs due to the limited computer resources, but it will be enough for a test study.

Subsequently, the recurrent selection schemes were performed for ten generations with 50 replicates independently among six scenarios (RAND, GS_HC_, GS_SL_, S1_SHI_, S2_SHI_ and S_DGI_). In each generation, according to the scenario-specific criteria, 20 sires and 20 dams were selected and crossed with a full-factorial mating system to create next-generation where each mating cross generated 20 progenies (total 8,000 progenies). Only 2,000 fish out of 8,000 progenies remained as the broodstock candidates for the next generation. This process was performed for a total of ten generations with 50 replicates. The broodstock population produced in the i-th generation was noted as F_i_ (i = 1,2,3…10). The scenario-specific criteria were as following. In the RAND scenario, parental individuals were randomly selected from the candidates (n = 2,000) in each generation. The individuals with high GEBVs for only a single trait were chosen in the GS_HC_ and GS_SL_ scenario, while the ones with high LGSIs in S1_SHI_, S2_SHI_ and S_DGI_ Scenario. For each generation, in GS_SL_ scenario, GBLUP model was trained using all candidates (n = 2,000) and broodfish were directly selected from these individuals, whilst, in GS_HC_, S_SHI_ and S_DGI_ scenario, GBLUP model was trained using half of the candidates (n = 1,000) and broodfish were selected from the remaining (n = 1,000) since fish should be sacrificed to obtain HC phenotype. The GBLUP model was implemented by *RRBLUP* function in R/AlphaSimR package.

**Reference**

1. Gaynor, R. C., Gorjanc, G. & Hickey, J. M. AlphaSimR: An R-package for Breeding Program Simulations. *bioRxiv* 2020.08.10.245167 Preprint at, https://doi.org/10.1101/2020.08.10.245167 (2020).
2. Kai, W. *et al.* Integration of the genetic map and genome assembly of fugu facilitates insights into distinct features of genome evolution in teleosts and mammals. *Genome Biol. Evol.* **3**, 424–442 (2011).
